# A Flavone-Based Solvatochromic Probe with A Low Expected Perturbation Impact on the Membrane Physical State

**DOI:** 10.3390/molecules25153458

**Published:** 2020-07-29

**Authors:** Simona Concilio, Miriam Di Martino, Anna Maria Nardiello, Barbara Panunzi, Lucia Sessa, Ylenia Miele, Federico Rossi, Stefano Piotto

**Affiliations:** 1 Department of Industrial Engineering, University of Salerno, 84084 Fisciano, Italy; 2 Department of Pharmacy, University of Salerno, 84084 Fisciano, Italy; miriamdimartino@hotmail.it (M.D.M.); annardiello@unisa.it (A.M.N.); lucsessa@unisa.it (L.S.); 3 Department of Agriculture, University of Napoli Federico II, 80055 Portici, Italy; barbara.panunzi@unina.it; 4 Department of Chemistry and Biology “A. Zambelli”, University of Salerno, 84084 Fisciano, Italy; ymiele@unisa.it; 5 Department of Earth, Environmental and Physical Sciences “DEEP Sciences”, University of Siena, 53100 Siena, Italy; federico.rossi2@unisi.it

**Keywords:** flavone, solvatochromic probe, membrane

## Abstract

The study of the cell membrane is an ambitious and arduous objective since its physical state is regulated by a series of processes that guarantee its regular functionality. Among the different methods of analysis, fluorescence spectroscopy is a technique of election, non-invasive, and easy to use. Besides, molecular dynamics analysis (MD) on model membranes provides useful information on the possibility of using a new probe, following its positioning in the membrane, and evaluating the possible perturbation of the double layer. In this work, we report the rational design and the synthesis of a new fluorescent solvatochromic probe and its characterization in model membranes. The probe consists of a fluorescent aromatic nucleus of a 3-hydroxyflavone moiety, provided with a saturated chain of 18 carbon atoms and a zwitterionic head so to facilitate the anchoring to the polar heads of the lipid bilayer and avoid the complete internalization. It was possible to study the behavior of the probe in GUV model membranes by MD analysis and fluorescence microscopy, demonstrating that the new probe can efficiently be incorporated in the lipid bilayer, and give a color response, thanks to is solvatochromic properties. Moreover, MD simulation of the probe in the membrane supports the hypothesis of a reduced perturbation of the membrane physical state.

## 1. Introduction

Biological membranes play an essential role in almost every cellular process and are characterized by a variety of lipid species, which differ in their physicochemical properties. The immiscibility of these components causes heterogeneity of the membrane and the formation of so-called “lipid rafts”. Rafts represent liquid-ordered domains (Lo). They consist mainly of saturated hydrocarbons (sphingolipids and phospholipids) and are free to move in the liquid disordered bilayer (Ld) of the membrane constituted by unsaturated fatty acids [1]. In recent years, there has been a growing interest in the study of membrane heterogeneity. Fluorescence techniques are one of the most widely used methods of characterization for membrane analysis, providing excellent tools for this purpose. These methods require the use of fluorescent probes, able to penetrate the membranes allowing a precise and immediate analysis [2,3]. The number of probes developed for the study of raft membranes is continuously increasing; those so far reported have restrictions related to the type of membrane and the fluorescence technique used [4,5,6].

**Scheme 1:**
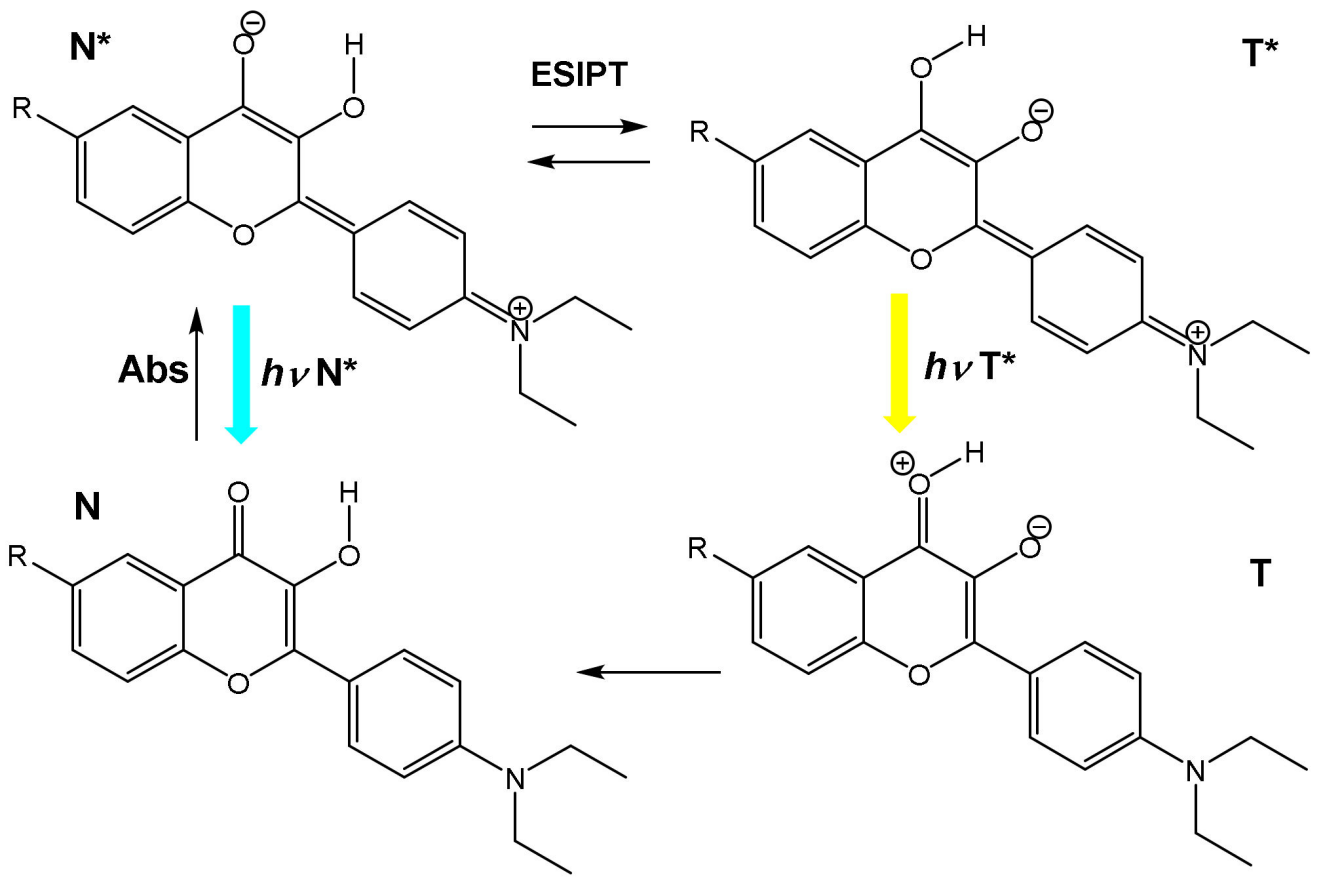
Diagram of ESIPT Reaction in 3HF.

The most recently identified class of probes corresponds to the class of environment-sensitive probes, which can directly distinguish the Lo and Ld phases due to differences in their intrinsic properties. These probes can change their fluorescence properties, fluorescence intensity, and even the emission color in response to changes in the environment [5,7,8,9]. This class includes solvatochromic fluorescent probes that show variations in their fluorescence properties depending on the polarity and hydration of the environment. Very promising in this context are the probes of the family of 3-hydroxyflavones (3-HF), in which an electron-donating moiety is present in position 4′. They show an excited state intramolecular proton transfer (ESIPT) reaction between the normal excited state N* and the tautomeric proton transfer product T*. The ESIPT process takes place by intramolecular proton transfer from the 3-hydroxy group to the 4-carbonyl (Scheme 1), thus resulting in two intense and well-separated emission bands [10,11].

The intensity and the position of the two emission bands depend on the solvent polarity. The states N* and T* have different charge distributions and interact differently with their environment. The N* form shows a high charge separation and, therefore, a higher dipole moment than the T* state. Proton transfer in the ESIPT process from the 3-hydroxy to the 4-carbonyl group causes a shift of the negative charge, reducing the polar character of the T* species. On the other hand, the presence of the electron-donating N,N-diethylamino group, contributes to the increase of the dipole moment of the species N* (this is not observed for the T* state) [12]. This difference in the distribution of charge results in a higher dependence of the N* band, located at shorter wavelengths, on the solvent polarity. An important parameter is the intensity ratio of emission maxima of the two bands I^N*^/I^T*^, which is directly correlated to the energy of the two states and provides an indication of the dependence on the solvent-polarity [13]. In aprotic solvents, the ESIPT process is speedy, because it is barrier-free, and this results in a decrease in intensity of the N* state band. In protic solvents, the solvation and formation of H-intermolecular bonds impose a high activation barrier that leads to a redshift of the N* band with an increase in intensity. In contrast, the fluorescence of the T* state is almost indifferent to the solvent polarity and remains practically unchanged [14,15]. This common effect in protic solvents, such as methanol and ethanol, sometimes results in the presence of a single band in the emission spectra, because of the overlap of the N* band, shifted at longer wavelengths, and T* band poorly resolved [16,17]. 

It is well known that the insertion of a probe into a bilayer can result in a, sometimes drastic, perturbation of the membrane physical state. [18,19,20]. This perturbation can impact the experimental results, so it is of primary importance to verify that the probe is capable to fit into the double layer without significantly changing its characteristics.

In this work, we report the design, synthesis, characterization, and the study in lipid vesicles and model membranes of a new solvatochromic probe for membrane analysis. The new probe, called 3HF18 and bearing the 3-hydroxyflavone moiety, has been provided with a saturated chain of 18 carbon atoms and a zwitterionic head so to facilitate the anchoring to the polar heads of the lipid bilayer and avoid the complete internalization, compared to similar existing probes [21]. The presence of the electron-donating N,N-diethylamino group on the 3-hydroxyflavone moiety, contributes to the increase of the dipole moment of the N* form, and ensures a high solvatochromic effect. Also, absorption and fluorescence analyses in organic solvents with different polarities were carried out to investigate the solvatochromic properties. Finally, the behavior of the probe 3HF18 in the model membrane was studied through DFT analysis and by fluorescence microscopy on GUVs.

## 2. Results and Discussion

### 2.1. Probe Design and Synthesis

**Figure 1:**
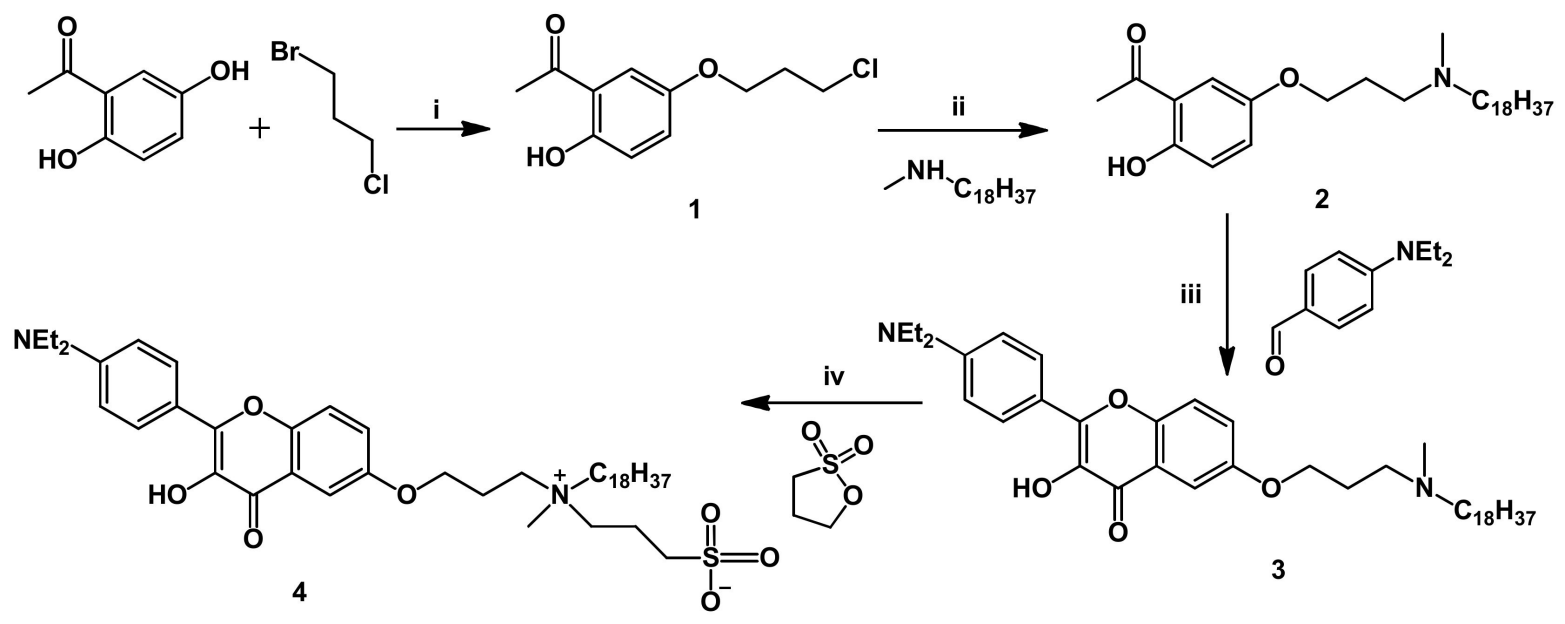
Synthetic scheme of the 3HF18 probe (**4**). *Reagents and Conditions*: (i) K_2_CO_3_, DMF dry, 80 °C, 4 h; (ii) KI, K_2_CO_3_, CH_3_CN dry, reflux, 48 h; (iii) (a) NaOMe, DMF dry, RT, 24 h; (b) EtOH, NaOMe, H_2_O_2_, reflux, 30 min; (iv) K_2_CO_3_, CH_3_CN dry, reflux 48 h.

The synthetic scheme of the probe 3HF18 (**4**) is illustrated in Figure 1.

Compound **1** was prepared according to a reported method [16]. The introduction of the C_18_ hydrocarbon chain was obtained by reaction with N-methyloctadecylamine in the presence of a base (step ii). Compound **2** was condensed with N,N-diethylaminobenzaldehyde under basic conditions, and then oxidized in the presence of hydrogen peroxide (step iii), so affording the fluorescent 3-hydroxyflavone core by oxidative cyclization (Algar–Flynn–Oyamada modified reaction) [22]. The quaternization of the amine by reaction with 1,3-propanesultone gave the fluorescent probe product 3HF18 (**4**) with a zwitterionic group. The products of all steps were purified by column chromatography or preparative TLC and fully characterized. NMR and mass spectrometry have confirmed the structure of the final product.

### 2.2. Optical Characterization

**Figure 2:**
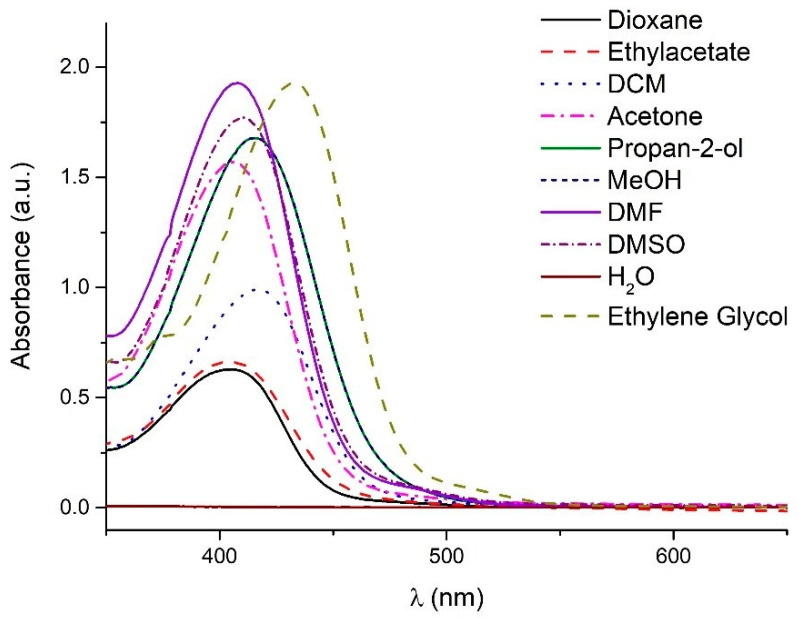
Absorption spectra of 3HF18 in solvents of different polarity.

**Figure 3:**
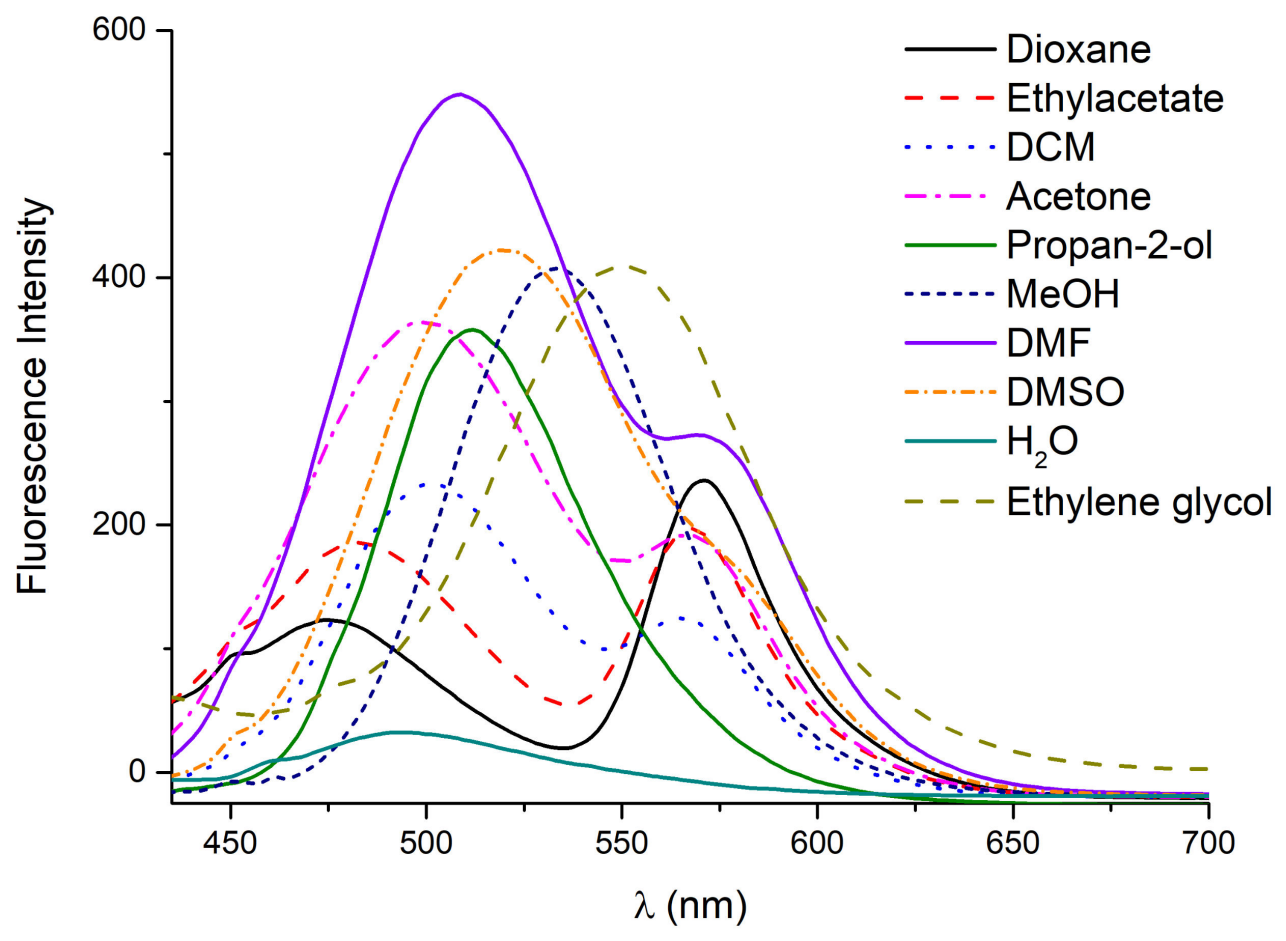
Emission spectra of 3HF18 in solvents of different polarity, excited at 398 nm.

The probe 3HF18 was analyzed by UV-Vis spectrophotometry and spectrofluorimetry in solvents with different polarity (Table 1, Figure 2 and Figure 3). The UV-Vis spectra show absorption maxima between 400 nm and 450 nm, with a redshift of more than 50 nm and an increase in absorbance as the polarity of the solvent increases. Emission spectra confirm the solvatochromic property of the probe in emission rather than in absorption. The probe has been shown to detect even slight changes in the polarity of the environment. In polar solvents both N* and T* are energy stabilized and this stabilization is more pronounced for N* due to the higher electrical dipole moment. For very polar protic solvents, N* is at energy lower than T*, and therefore only one transition is observed (Figure 3). Moreover, as reported in the literature for analogous compounds, the probe’s core is poorly soluble and nonfluorescent in water [23]. The solubility increases in water/methanol mixtures. In these cases, the fluorescence spectra show a single fluorescence peak around 520 nm (Figure S5 in Supplementary Materials). As can be observed in Figure 3, the maximum emission ranges from 475 nm in dioxane to 551 nm ethylene glycolfor the N* band. While the T* band, located at longer wavelengths, shows almost no influence with the solvent polarity because the stabilization effect of polar solvents acts on both T and T* states. The I^N*^/I^T*^ ratio represents the dependence of the fluorescence intensity on the solvent polarity. It is possible to observe an increase in the intensity of fluorescence, which accompanies the redshift, changing from apolar solvents such as dioxane to a single band (N* state) in protic polar solvents such as methanol, propan-2-ol and ethylene glycol.

**Figure 4:**
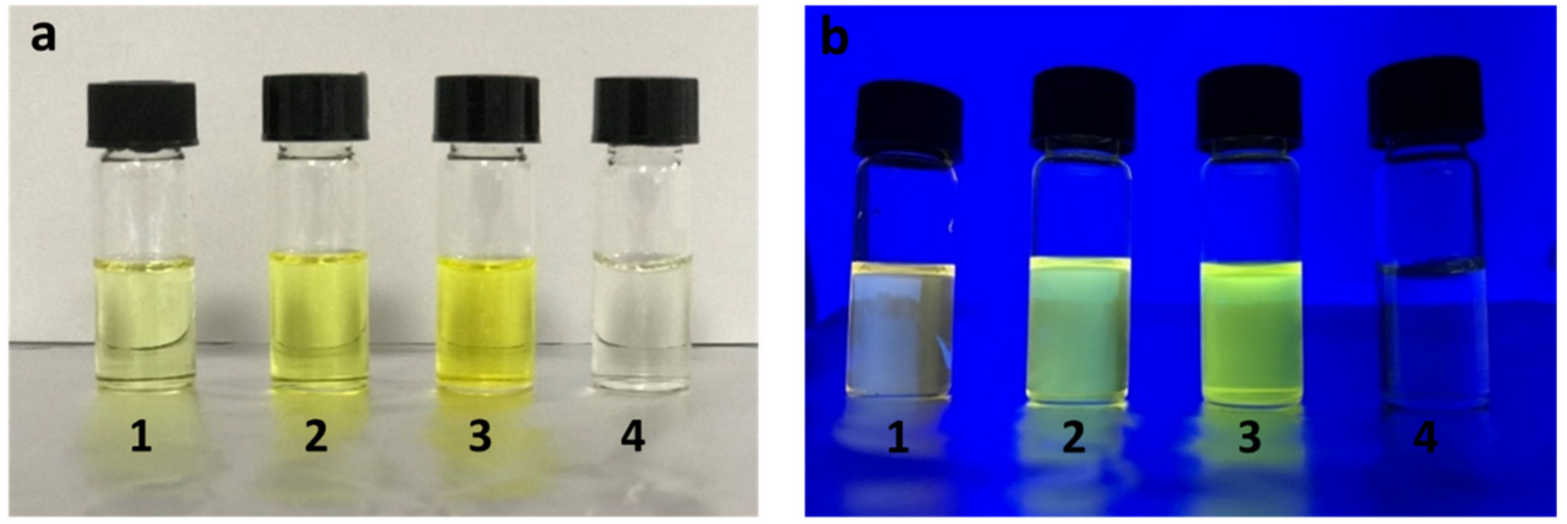
Picture of 3HF18 dissolved in different representative solvents: (1) Dioxane, (2) DCM, (3) MeOH, (4) H_2_O: (**a**) under white light and (**b**) under UV light (365 nm).

In Figure 4, a picture of different solutions of the probe 3HF18, in representative solvents with increasing polarity is reported, under white (a) and UV (b) light.

From the image in Figure 4, the solvatochromism of the 3HF18 molecule is visible to the naked eye. The emission color of the solutions ranges from orange to yellow-green, as the polarity of the solvent increases. The aqueous solution appears colorless due to the poor solubility of the compound.

### 2.3. Analysis of Fluorescence in Lipid Membranes

**Figure 5:**
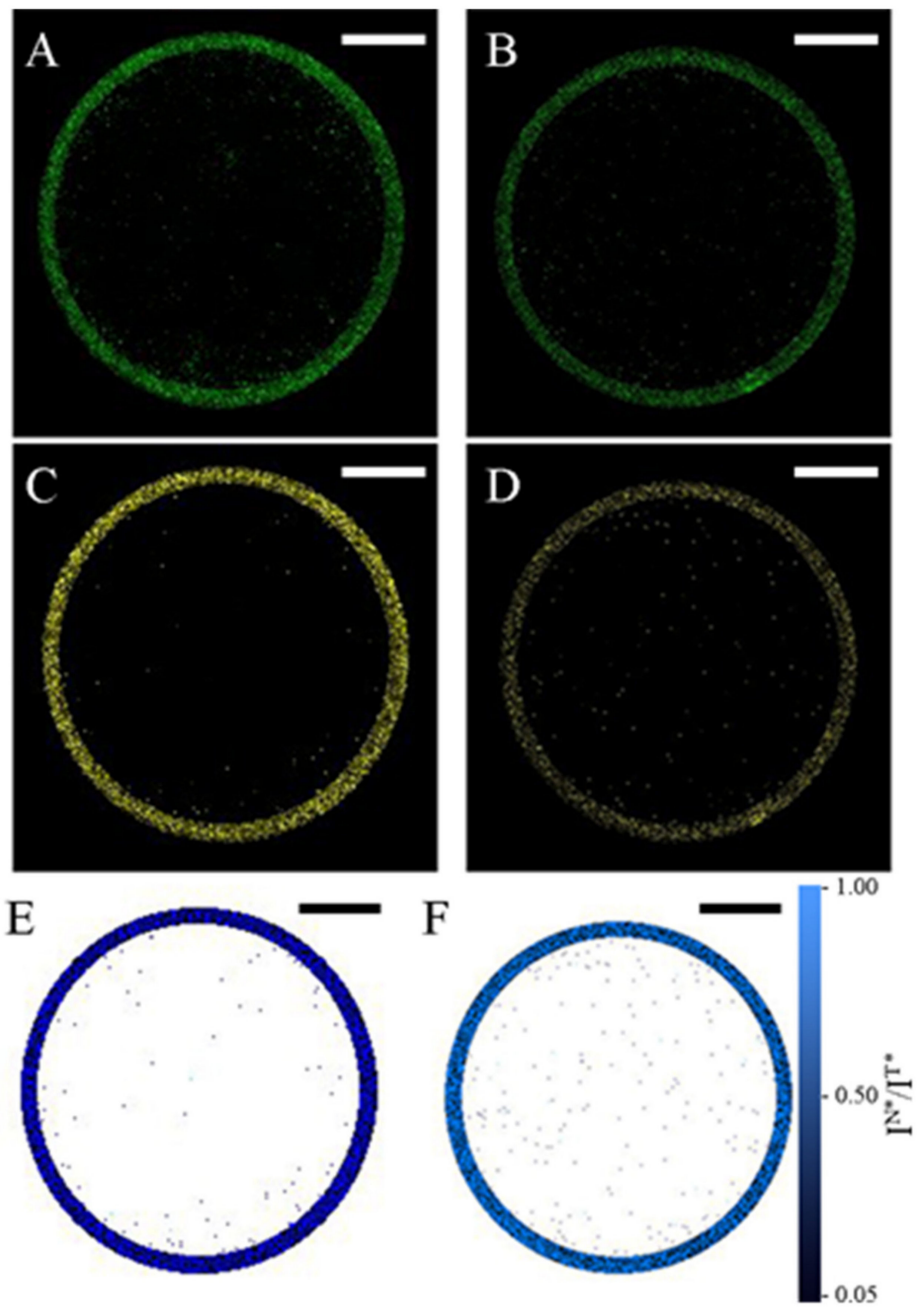
Fluorescence confocal images (**A**–**D**) and intensity ratio (**E**,**F**) of vesicles made of POPC (**A**,**C**,**E**) and POPC/DPPC/Chol 1/0.5/0.7 (**B**,**D**,**F**). Excitation at λ = 405 nm, Emission at λ = 477–527 nm (**A**,**B**) and λ = 550–600 nm (**C**,**D**). Bars scale 5 μm. Contrast of the images has been adjusted to improve the quality of the pictures.

The heterogeneity and dynamism of cell membranes make it challenging to study their structure because they are incredibly complex entities. For this reason, the simplest model for membranes is represented by unilamellar vesicles. In particular, giant unilamellar vesicles, GUVs, with dimensions comparable to the sizes of a cell (in an interval of about 1–100 µm), are particularly useful as model membranes. Over the years, several methods have been developed for the design of GUVs. Many methods have been proposed to control the size and composition of the vesicles [24,25]. One of the most recently developed methods is the “droplet transfer” or “reverse emulsion” [26], characterized by the stratification of an aqueous phase and a water-in-oil emulsion with the formation of the bilayer at the interface of the two phases. This method permits to easily encapsulate hydrophilic compounds in the aqueous lumen [27,28] and blend hydrophobic species in the membrane, such as fatty acids [29,30] or polymers [31,32]. In this work, the “droplet transfer” method was used for the formation of vesicles of POPC and a ternary mixture of POPC:DPPC:Chol to simulate the two phases Lo and Ld of a membrane. The solvatochromism of the probe 3HF18 can be used to characterize cell membranes or their simplified models consisting of lipid vesicles. The probe has been successfully encapsulated both in pure POPC vesicles in the Ld phase, Figure 5A,C and in mixed POPC:DPPC:Chol membranes (Lo phases, Figure 5B,D). Fluorescence has been recorded at two different wavelength intervals, where the emission maxima do not overlap: in (Figure 5A,B) λ_exc_ = 405 nm, the emission range is λ = 477–527 nm; in Figure 5C,D λ_exc_ = 405 nm and the emission range is λ = 550–600 nm. Cholesterol is reported to increase polarity along the membrane surface, while in the hydrophobic core it decreases polarity [33]. In the case of mixed vesicles, therefore, due to the effect of Chol, the surface polarity may be higher than in vesicles of POPC alone. In a previous work [21], we have shown that membrane probes with a zwitterionic head can be easily placed with the chromophore beneath the membrane surface and with the hydrophobic tail inserted between the tails of phospholipids. In this case, a higher I^N*^/I^T*^ emission ratio in GUVs of POPC/DPPC/Chol is observed (Figure 5E,F) which supports the notion that the chromophore is positioned below the surface of the membrane.

The fluorescence of the probe is limited to the surface of the vesicles, thus confirming the anchorage of the probe to the double layer. Molecular dynamics studies indicate that the flip flop mechanism of the probe is significantly lower than that of fluorophore alone and also than that of POPC and DPPC. In fact, the zwitterionic head of the probe has a dipole inverted compared to that of phospholipids and increases its membrane cohesion.

### 2.4. Molecular Dynamics in Model Membranes. Thickness and Area Per Lipid

Through molecular dynamics (MD) analysis, double layer thickness and area per lipid values were calculated. The experimental values of double layer thickness and area per lipid (A_l_) are commonly used for the validation of simulations [34,35]. The average lipid bilayer thickness, calculated as the average distance between the phosphorus atoms of the two double-layer sheets, showed that the insertion of the probe, in both excited N* and T* forms, does not cause membrane perturbation (Table 2).

The thickness values obtained from pure membrane simulations are 37.3 Å for POPC and 45.9 Å for DPPC-Chol. The calculated thickness values are in good agreement with the experimental values of 39.8 ± 0.8 Å [35] for POPC and 44.8 Å for DPPC-Chol [34], respectively. From the values shown in Table 2, it can be seen that the inclusion of the 3HF18 molecule does not cause a change in the thickness of the analyzed membranes. Table 3 shows the area per lipid (A_l_) data obtained from simulations on different systems compared with the experimental data obtained from CHARMM-GUI [36].

When the cholesterol concentration inside the membrane is high (>20%), it is necessary to decrease the area per lipid values by 10–20%. Therefore DPPC-Chol displays a range of values. Also, for this analysis, the areas per lipid in the presence and absence of the probe are comparable, so we can conclude that the presence of the probe does not cause perturbation in the membranes.

### 2.5. Deuterium Order Parameter SCD

**Figure 6:**
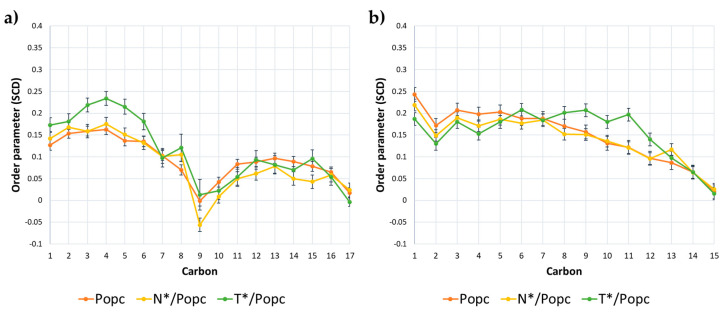
Order parameter SCD for (**a**) the unsaturated oleic and (**b**) the saturated palmitoyl acyl chains of POPC phospholipid in POPC pure membrane (orange curve) used as control and in POPC/3HF18 membrane (N*, yellow curve; T* green curve). Notes: On the *Y*-axis, the SCD is indicated; on the *X*-axis, the carbon atom position is reported, starting from the first (1) alpha carbon atom in the chains.

**Figure 7:**
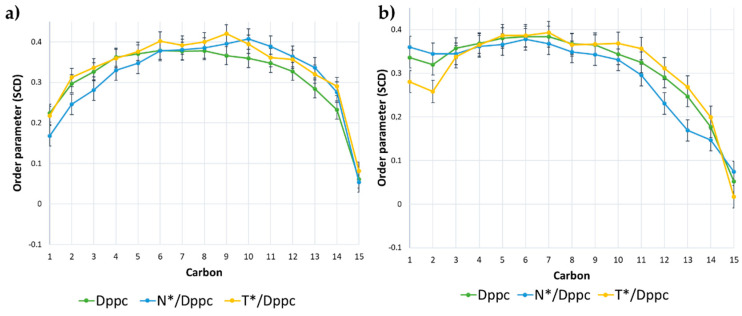
Order parameter SCD for (**a**) the saturated Sn-1 palmitoyl chain and (**b**) the Sn-2 saturated palmitoyl chains of DPPC phospholipid in DPPC pure membrane (green curve) used as control and in DPPC/3HF18 membrane (N*, blue curve; T* yellow curve). Notes: On the *Y*-axis, the SCD is indicated; on the *X*-axis, the carbon atom position is reported, starting from the first (1) alpha carbon atom in the chains.

To assess whether the probe causes a change in membrane fluidity, we performed an analysis of the deuterium order parameter (SCD. The SCD parameter is a measure of the motor anisotropy of the C-D bond analyzed and provides its orientation over time. It is derived from NMR experiments. SCD analysis provides comprehensive information on membrane fluidity and allows us to predict changes in the fluidity of the lipophilic chains of phospholipids near a guest molecule (in this case, the 3HF18 probe). The fluidity of the POPC and DPPC-Chol chains was evaluated in the presence and absence of the 3HF18 probe in the forms N, N* and T* (Figure 6 and Figure 7). The results of the N-form calculations are reported in the Supplementary Materials section. In Figure 6, the SCDs for POPC phospholipid chains around 5 Å from the guest molecule is shown. In Figure 6a, the unsaturated chain is shown, and in Figure 6b, the saturated one.

The SCD decreases sharply in the region near the double bond (carbon atom 9) and approaches 0 in the tail region, indicating the highest disorder within the double layer (Figure 6). Similar SCD profiles were observed experimentally using NMR studies [37]. Figure 7 shows the SCD values of the two saturated chains of DPPC. Even for the DPPC, in the final region of the tail, the SCD decreases towards 0, thus showing a greater disorder.

From Figure 6 and Figure 7, we can see that the probe (both tautomeric forms of 3HF18) interacts with the aliphatic chains of phospholipids and does not cause a significant change in the order of the membranes.

### 2.6. Pseudo-Semantic Analysis

**Figure 8:**
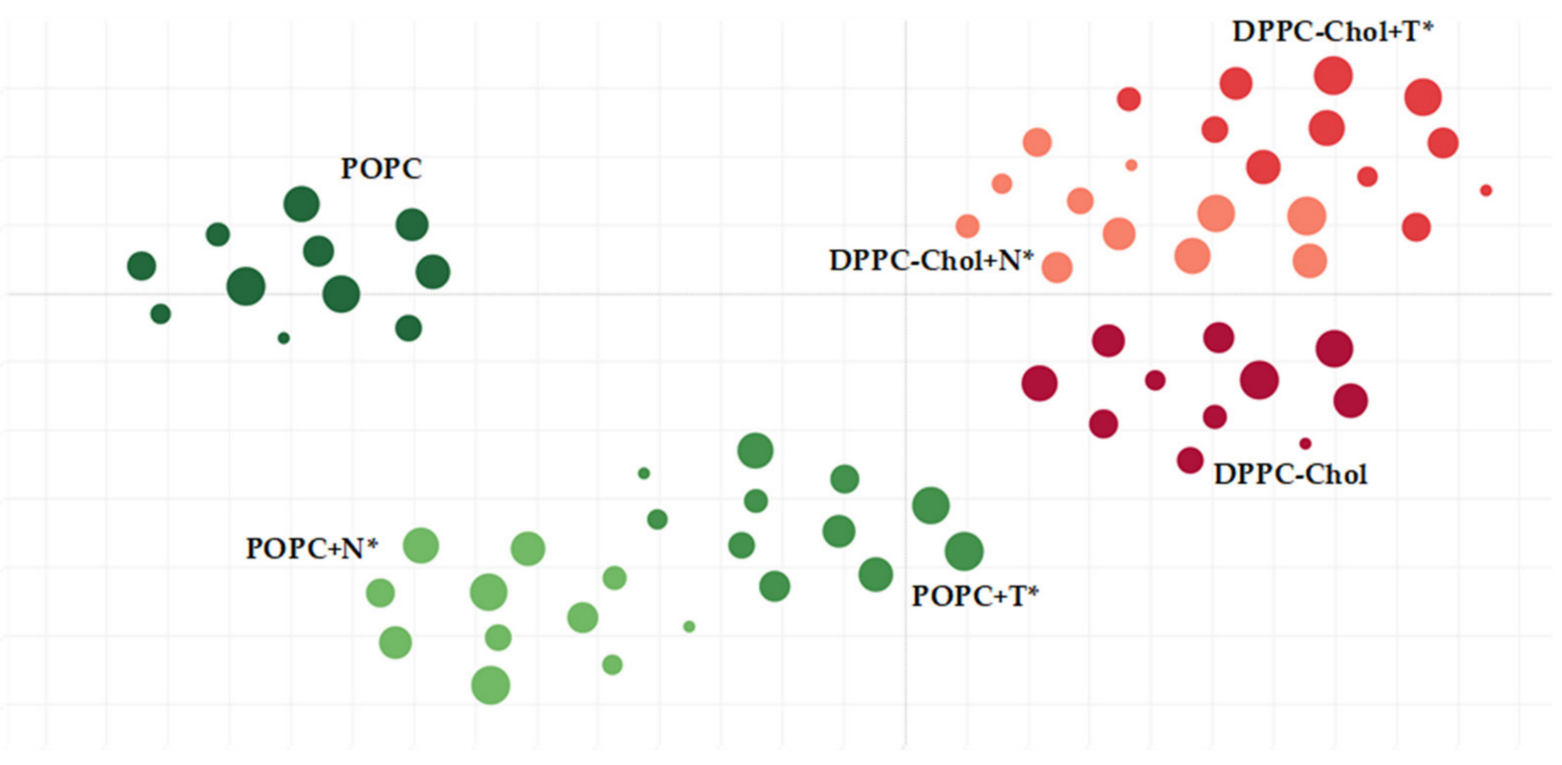
t-SNE dispersion graph. Each point corresponds to a frame of molecular dynamics. Circle dimension is related to the time (ns). Green circles: POPC membranes; red/orange: DPPC-Chol systems.

A new approach to evaluate membrane fluidity is to evaluate SCD by pseudo-semantic analysis. The SCD parameter, obtained from MD analysis, is converted into alphabetical strings, which are compared and clustered by similarity. The advantage compared to the previous analysis, is the possibility to compare the different membrane/probe systems and to analyze the fluidity of the individual frames during a simulation. The result, displayed through a dispersion graph, is shown in Figure 8.

In Figure 8, the six systems analyzed are shown. Each simulation of MD generates 11 frames, whose dimensions increase with the time of the simulation (ns). Each circle corresponds to one of the 11 frames of the trajectory. In different shades of green are shown the systems in POPC, and in red/orange the systems in DPPC-Chol. The analysis is capable to capture the similarity among different membranes at different times. Figure 8 shows that DPPC-Chol membranes are rather similar, forming almost a single cluster (top right). This fact indicates that the presence of the probe does not significantly perturb the membrane and that the order of the aliphatic chains during the whole dynamics remains similar. On the contrary, POPC membrane (and reasonably any kind of Ld membranes), are slightly influenced by the presence of the probe. The ordering effect of the probe is revealed by the change in the position of the POPC + T* cluster toward the DPPC-Chol cluster.

### 2.7. The Binding Energy Between Probe and Membrane

In order to check whether either tautomeric form has a preference for the Lo or Ld phase of the membranes, the binding energy with the membrane/water system has been calculated. The binding energies for the last 5 ns of the simulation are shown in Table 4.

**Figure 9:**
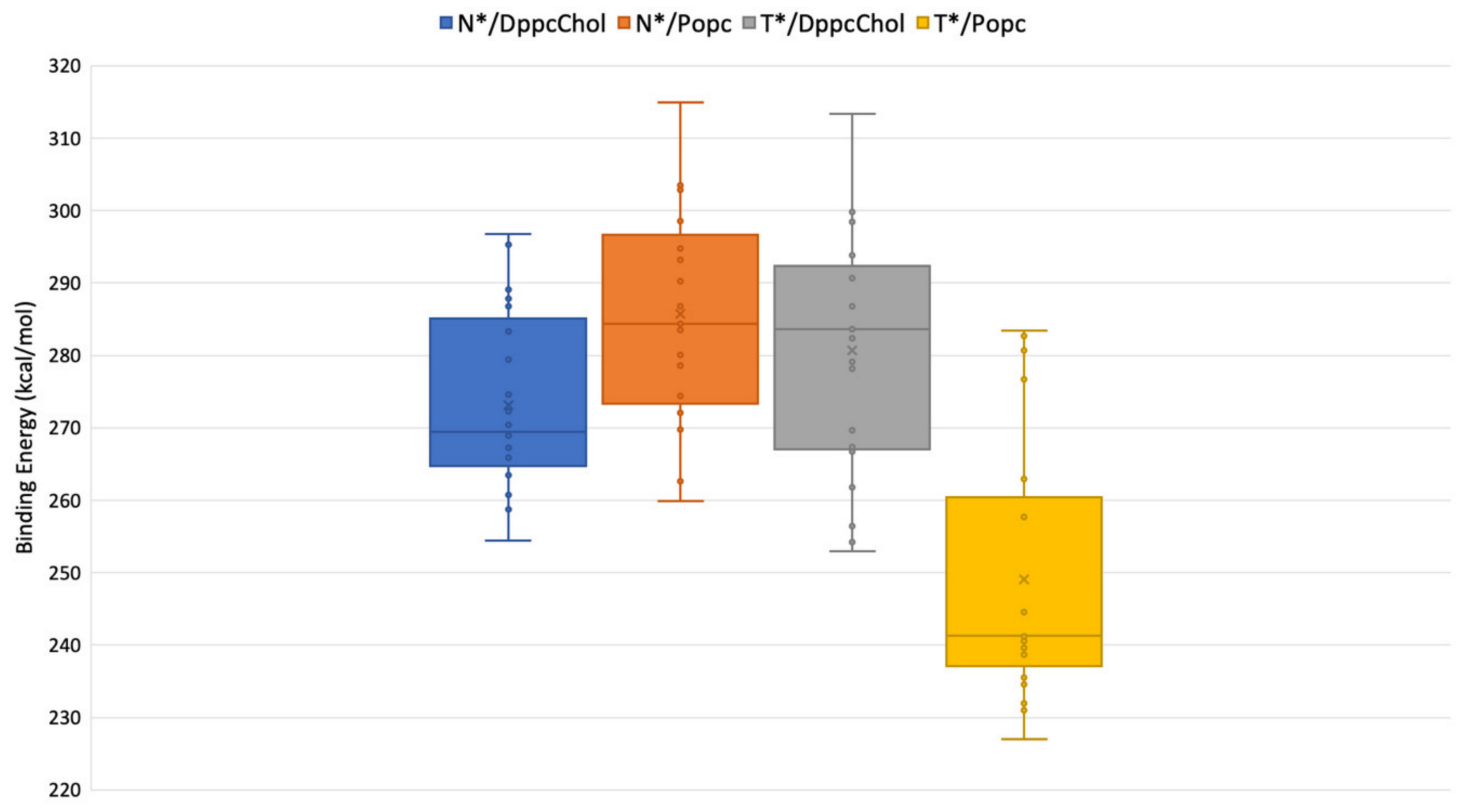
Box plot of binding energies on four systems: the 3HF18 probe in the N* form in DPPC-Chol membrane (blue); the 3HF18 probe in the N* form in POPC membrane (orange); the 3HF18 probe in the T* form in DPPC-Chol membrane (grey); the 3HF18 probe in the T* form in POPC membrane (yellow). The binding energy expressed in kcal/mol is shown on the *y*-axis.

As we can see from Table 4 and Figure 9, each tautomeric form has a preference for a membrane: N* has better interaction with the POPC membrane while T* interacts better with DPPC-Chol. To validate the energy binding results, a t-test statistical analysis was performed for the different POPC and DPPC systems with the two forms of the 3HF18 probe, and the results are reported in the Supplementary Materials section. The analysis shows that the energies are significantly different, with a probability of statistical significance of over 98%.

## 3. Materials and Methods

All the reagents and solvents were purchased from Sigma Aldrich (Milan, Italy) and used without further purification. Optical observations of the probe in GUVs were performed by using a TCS SP8 Confocal laser (Leica, Wetzlar, Germany), UV laser λ = 405 nm, emission range for the N* transition λ = 477–527 nm, emission range for the T* transition λ = 550–600 nm). The images were acquired with a sequential scan between lines and an average of three frames. UV–vis absorption spectra of the samples were recorded at 25 °C in acetonitrile solution, on a Lambda 800 spectrophotometer (Perkin Elmer, Rodgau, Germany). The spectral region 650–240 nm was investigated by using a cell path length of 1.0 cm. Probe concentration of about 3.0 × 10^−5^ mol L^−1^ was used. Fluorescence spectra were performed at 25 °C in acetonitrile solution, on a Jasco FP-750 Spectrofluorometer (JASCO Europe, Cremella (LC) Italy), at a concentration of about 3.0 × 10^−7^ mol L^−1^. ^1^H-NMR spectra were recorded with a DRX/400 spectrometer (Bruker, Billerica, MA, USA). Chemical shifts are reported relative to the residual solvent peak (chloroform-d: H = 7.26 ppm). The following abbreviations are used to express spin multiplicities in ^1^H NMR spectra: s = singlet; d = doublet; dd = double doublet; t = triplet; m = multiplet. High-resolution mass spectra were acquired on an LTQ-Orbitrap instrument (Thermo-Fisher, Waltham, MA, USA) operating in positive ion mode. The probe was dissolved in methanol at a concentration of 0.1 mg/mL and injected into the MS ion source. Spectra were acquired in the 150–800 *m*/*z* range.

### 3.1. Synthesis

The probe 3HF18 was synthesized according to the following procedure.

#### 3.1.1. Synthesis of 5-(3-chloropropoxy)-2-hydroxy-acetophenone (**1**)

2,5-Dihydroxyacetophenone (2.0 g, 0.013 mol) was dissolved in dry DMF (13 mL). Then, K_2_CO_3_ (1 eq, 1.8 g, 0.013 mol) and 1-bromo-3-chloropropane (1.1 eq, 1.4 mL, 0.0145 mol) were added. The reaction mixture was stirred under a nitrogen atmosphere heating at 80 °C for 4 h. The solution became dark. After cooling, it was diluted with water and extracted with ethyl acetate (three times). The combined organic layers were dried over anhydrous sodium sulfate, filtered and the solvent evaporated under reduced pressure. The obtained dark oil was purified by flash column chromatography in silica gel (hexane: ethyl acetate, 98:2) to produce compound **1** as a light-yellow solid (1.13 g, 0.005 mol). Yield 38%. ^1^H-NMR (400 MHz, CDCl_3_): δ 2.16 (m, 2H), 2.55 (s, 3H), 3.70 (t, 2H), 4.03 (t, 2H), 6.86 (1, 2H), 7.05 (dd, 1H), 7.14 (d, 1H).

#### 3.1.2. Synthesis of 5-(3-(methyl(octadecyl)amino)propoxy)-2-hydroxyacetophenone (**2**)

5-(3-Chloropropoxy)-2-hydroxyacetophenone (**1**, 1.0 g, 0.0044 mol) was dissolved in dry acetonitrile (55 mL) and KI (1.5 eq, 1.095 g, 0.0066 mol), K_2_CO_3_ (2.5 eq, 1.52 g, 0.011 mol) and N-methyloctadecylamine (1.2 eq, 1.50 g, 0.0053 mol) were added to the solution. The reaction mixture refluxed under stirring in a nitrogen atmosphere for 48 h. After cooling, the solution was extracted with chloroform three times. The organic layer was dried over anhydrous sodium sulfate, filtered, and the solvent was evaporated under reduced pressure. The residue was purified by flash column chromatography in silica gel (dichloromethane: methanol, 95:5) to produce the desired product 2 as a brown solid (1.530 g, 0.0033 mol). Yield 74%. ^1^H-NMR (400 MHz, CDCl_3_): δ 0.91 (t, 3 H), 1.27 (s, 32 H), 2.10 (q, 2 H), 2.43 (s, 3 H), 2.58 (t, 2 H), 2.63 (s, 3H), 2.76 (t, 2 H), 4.04 (t, 2 H), 6.92 (d, 1 H), 7.11 (dd, 1 H), 7.21 (d, 1 H).

#### 3.1.3. Synthesis of 4′-diethylamino-6-(3- methyloctadecylaminopropoxy)-3-hydroxyflavone (**3**)

5-(3-(Methyl(octadecyl)amino)propoxy)-2-hydroxyacetophenone (**2**, 800 mg, 1.68 mmol) was dissolved in DMF (10.0 mL) and then 4-diethylaminobenzaldehyde (1.05 eq, 313 mg, 1.76 mmol) and sodium methoxide (4.0 eq, 363.0 mg, 6.72 mmol) were added. The obtained mixture was stirred for 24 h to give dark red chalcone, which was used in the next step without isolation. This mixture was then diluted with 10.0 mL of ethanol and then sodium methoxide (12 eq., 1.088 g, 20.16 mmol) was added. The temperature was reduced to 0 °C to add hydrogen peroxide (10 eq, 1.9 mL). This mixture was kept refluxing for 30 min under stirring and the dark red solution turned orange. The solution was cooled at room temperature, diluted with water and neutralized with 10% hydrochloric acid. The product was then extracted with ethyl acetate. The solvent was removed under vacuum and the crude product was purified by flash column chromatography in silica gel (dichloromethane: methanol, 90:10) to give the desired product **3** as a yellow solid (148 mg, 0.228 mmol). Yield 13.5%. ^1^H-NMR (400 MHz, CDCl_3_): δ 0.8 (t, 3H), 1.05 (t, 6 H), 1.2 (s, 30 H), 1.95 (q, 2 H), 2.35 (q, 2 H), 2.75 (s, 3 H), 2.95 (t, 2 H), 3.2 (t, 2 H), 3.38 (q, 4 H), 4.1 (t, 2 H), 6.7 (d, 2 H), 7.15 (dd, 1H), 7.41 (d, 1 H), 7.45 (d, 1 H), 8.07 (d, 2 H).

#### 3.1.4. Synthesis of N-[3-(4′-diethylamino-3-hydroxyflavonyl-6-oxy)propyl]-N,N-(methyloctadecyl ammonium) propane-1-sulfonate (**4**)

4′-Diethylamino-6-(3- methyloctadecylaminopropoxy)-3-hydroxyflavone (**3**, 90 mg, 0.138 mmol) was dissolved in dry acetonitrile (10 mL) and 1,3-propanesultone (2.5 eq, 42.0 mg, 0.345 mmol) and K_2_CO_3_ (1 eq.,19.0 mg, 0.138 mmol) were added. The reaction was stirred at reflux under nitrogen atmosphere for 48 h. The solvent was evaporated under reduced pressure and the crude was purified by preparative TLC (dichloromethane: methanol, 85:15) to give product **4** as a yellow solid (25 mg; 0.032 mmol). Yield 24%. ^1^H-NMR (400 MHz, MeOD): δ 0.91 (6 H, t), 1.23-1.36 (33 H, m), 1.79 (2 H, m), 2.25 (2 H, m), 2.34 (2 H, m), 2.91 (2 H, t), 3.13 (3 H, s), 3.52 (4 H, m), 3.59 (4 H, m), 4.27 (2 H, t), 6.85 (2 H, d), 7.41 (1 H, dd), 7.59 (1 H, d), 7.65 (1 H, d), 8.22 (2 H, d). ^13^C-NMR (600 MHz, MeOD): δ 11.50, 13.01, 18.17, 21.26, 21.87, 22.31, 25.94, 28.78, 29.05, 29.13, 29.24, 29.37, 31.65, 48.16, 58.46, 60.39, 61.64, 64.70, 78.04, 104.71, 119.46, 121.76, 123.05, 129.44, 136.87, 150.11, 155.06, 172.16. TOF-MS (ES+) calcd for C_44_H_70_N_2_O_7_S + Na^+^: 793.48; found [M + Na]^+^: 793.58.

### 3.2. Preparation of GUVs

GUVs were made either with pure POPC or with a mixture POPC:DPPC:Chol = 1:0.5:0.7, in both cases the preparation method was the same. Here we report only the one for the mixed membrane. In brief, an Eppendorf tube (1.5 mL) was filled with: (*i*) an “outer solution” (0.5 mL of water, 200 mM glucose); (*ii*) an interfacial phase (0.3 mL, 0.3 mM POPC in mineral oil, 0.15 mM of DPPC in mineral oil, 0.21 mM of Chol in mineral oil); (iii) a water/oil emulsion (0.6 mL) prepared by pipetting up and down the “inner phase” made mixing 20 μL of water (200 mM sucrose) with an hydrophobic phase. The hydrophobic phase was prepared by mixing POPC (0.3 mM), DPPC (0.15 mM), Chol (0.21 mM) in mineral oil and adding the 3HF18 probe (4 µM) first dissolved in chloroform and subsequently dried after the evaporation of the solvent. The tube was immediately centrifuged (6000 rpm, 10 min, RT). After centrifugation, mineral oil was removed, and GUVs were washed twice to remove free solutes by pelleting/resuspension. 30 μL of pellet was resuspended in 20 μL of glucose (200 mM). 20 μL of solution was put on a microscope glass slide and observed with confocal microscopy. Typically, GUVs had diameters between 12–50 μm [26,27,29,38].

### 3.3. Molecular Dynamics Simulations

All MD simulations were performed with YASARA Structure v19.12.14 [39]. The force-field used is AMBER15FB under NPT (normal pressure and temperature) conditions, coupling the system to a Berendsen thermostat and using a combined solvent pressure and density control. The applied cut-off is 8 Å, and the Particle Mesh Ewald (PME) model has been used to calculate the electrostatic interactions, as it has been demonstrated that the correct treatment of electrostatic is essential in biological membrane systems. The systems are fully hydrated (water density 0.997 g/mL). The simulation cell was neutralized with NaCl, with a final concentration of 0.9%. After membrane equilibration, the probe was incorporated by positioning the aliphatic chain perpendicular to the bilayer [40,41,42]. MD simulations lasted 15 ns, of which only the last 5 ns were used for analysis. With the same MD parameters, the binding energy between the 3HF18 probe and the hydrated membranes was also calculated.

### 3.4. Model Membrane Structural Parameters

The structural parameters of the membrane are the thickness, the fluidity of the aliphatic chains, and the area per lipid. The last parameter has been calculated by dividing the surface area of the membrane by the number of lipids that compose it. The lipid area was then compared with the experimental membrane values reported in CHARMM-GUI [36]. The fluidity was measured indirectly by calculating the deuterium order parameter (SCD) with the VMD MembPlugin software [43,44].

### 3.5. Pseudo-Semantic Approach for the Study of Membrane Fluidity

A new approach for the study of membrane fluidity [45,46] consists of converting the results obtained from the dynamics into alphabetical strings, which are then compared and clustered by similarity. The parameter examined, in this case, is the SCD calculated using VMD MembPlugin [44]. The last 5 ns of simulation correspond to 11 frames. For each frame, the SCD values of phospholipids within 5 Å of the probe are taken into account. Each SCD value is assigned a letter. A total of 66 alphabetical strings are produced (11 for each system). In each string, the SCD values of each carbon making up the aliphatic chain of phospholipids under investigation are given in letters. The strings were then processed with Sysa (www.softmining.it/sysa), an online tool able to analyze the similarities between them. Sysa pre-processes the input files in order to eliminate redundant data and then extract user-defined k-mer length sub-strings. The metric used in this study is Szymkiew Simpson Coefficient with k-mer between 2 and 8. At the end of the computation, Sysa generated a matrix of distances. Due to the difficulty of displaying a matrix with more than three dimensions, we decided to process it using t-SNE (t-distributed stochastic neighbor embedding). t-SNE is a two-dimensional matrix dimensionality reduction algorithm [47].The algorithm allows keeping objects that were already close to each other in the original space at reduced dimensionality.

## 4. Conclusions

A novel fluorescent molecule was designed, synthesized, and optically characterized as a probe for lipid membranes. From the optical and confocal microscopy analysis of GUVs loaded with the probe, the probe is able to anchor to the lipid double layer, without internalizing. In addition, the fluorescence of the 3HF18 probe is visible in both Ld and Lo vesicles. MD simulations show that the insertion of the probe does not disturb the membrane, since both the membrane thickness and the order parameter remain essentially unchanged. Furthermore, thanks to a new semi-semantic approach, it was possible to assess that Ld membranes are more sensitive to probe insertion, and that the 3HF18 molecule shows a preference for the Lo or Ld phases of a model membrane, depending on the most stable tautomeric form.

## Figures and Tables

**Table 1:** Absorption and emission data of 3HF18 in solvents with different polarities.

Solvent	λ_Abs_ (nm) ^1^	ε (M^−1^ cm^−1^) ^2^	λ^N*^_Em_ (nm)	λ^T*^_Em_ (nm)	I^N*^/I^T*^
Dioxane	405	1.1⋅10^4^	475	571	0.48
AcOet	406	1.3⋅10^4^	483	568	0.94
DCM	416	2.4⋅10^4^	502	564	1.8
Acetone	426	3.2⋅10^4^	502	567	1.9
Propan-2-ol	440	3.4⋅10^4^	515	- ^3^	- ^3^
MeOH	440	3.4⋅10^4^	534	- ^3^	- ^3^
DMF	438	4.4⋅10^4^	509	576	2.05
DMSO	440	4.2⋅10^4^	519	- ^4^	- ^4^
Ethylene Glycol	453	4.4⋅10^4^	551	- ^3^	- ^3^
H_2_O ^5^	-	-	-	-	-

^1^ Maximum absorption wavelength; ^2^ Molar extinction coefficients, from Lambert-Beer Law; ^3^ A single N* peak is observed; ^4^ The T* band, poorly resolved, appears as a shoulder; ^5^ The probe is poorly soluble in water. For the emission spectra, all the solutions were excited at 398 nm.

**Table 2:** Thickness values (in Å) of the two membranes in the presence and absence of the probe.

POPC ^1^	DPPC-Chol ^1^	POPC ^2^	DPPC-Chol ^2^
37.3 ± 0.3	45.9 ± 0.2	39.8 ± 0.8	44.8
POPC + 3HF18(N*) ^3^	POPC + 3HF18(T*) ^4^	DPPC-Chol + 3HF18(N*) ^5^	DPPC-Chol + 3HF18(T*) ^6^
37.4 ± 0.2	37.8 ± 0.2	46.4 ± 0.3	46.1 ± 0.2

^1^ Thickness of simulated POPC and DPPC-Chol membranes; ^2^ Experimental thickness of POPC and DPPC-Chol membranes; ^3^ Thickness of POPC membrane with N* 3HF18; ^4^ Thickness of POPC membrane with the T* 3HF18; ^5^ Thickness of DPPC-Chol membrane with N* 3HF18; ^6^ Thickness of DPPC-Chol membrane with T* 3HF18.

**Table 3:** Area per Lipid (A_l_) values of the two membranes with and without the probe 3HF18.

POPC ^1^	DPPC-Chol ^1^	POPC + 3HF18 (N*) ^2^	POPC + 3HF18 (T*) ^3^	DPPC-Chol + 3HF18 (N*) ^4^	DPPC-Chol + 3HF18 (T*) ^5^
68.3	43.2-48.6	69.9 ± 0.5	69.2 ± 0.5	43.2 ± 0.2	43.6 ± 0.2

^1^ Experimental A_l_ of POPC e DPPC-Chol membranes; ^2^ A_l_ of POPC membrane with N* 3HF18; ^3^ A_l_ of POPC membrane with T* 3HF18; ^4^ A_l_ of DPPC-Chol membrane with N* 3HF18; ^5^ A_l_ of DPPC-Chol membrane with T* 3HF18.

**Table 4:** Binding energy values for the 3HF18 (form N* and T*) in POPC and DPPC-Chol membrane.

	POPC + 3HF18(N*) ^1^	POPC + 3HF18(T*) ^2^	DPPC-Chol + 3HF18(N*) ^3^	DPPC-Chol + 3HF18(T*) ^4^
Binding energy (kcal/mol)	286 ± 14	249 ± 18	273 ± 12	281 ± 16

^1^ The binding energy of the 3HF18 probe in POPC membrane N* form; ^2^ Binding energy of the 3HF18 probe in POPC membrane T* form; ^3^ Binding energy of the 3HF18 probe in DPPC-Chol membrane N* form; ^4^ Binding energy of the 3HF18 probe in DPPC-Chol membrane T* form.

## Supplementary Materials

The following are available online, Table S1: Thickness values (in Å) of the two membranes with and without the probe, Figure S1: SCD of the unsaturated POPC chain with (blue) and without (green) the probe, Figure S2: SCD of POPC saturated chain with (blue) and without (green) probe, Figure S3: SCD of the DPPC Sn-1 palmitoyl chain (DPPC-Chol) with and without probe, Figure S4: SCD of the DPPC Sn-2 palmitoyl chain (DPPC-Chol) with and without probe, Figure S5: Fluorescence spectra of probe 3HF18 in binary mixture MeOH/H_2_O in different ratios, Table S2: Results of t-Student test.
