# Supplementary material for: A Flavone-Based Solvatochromic Probe with A Low Expected Perturbation Impact on the Membrane Physical State
Source: Molecules. 2020 Jul 29;25(15):3458. doi: 10.3390/molecules25153458 (PMC7436088; doi:10.3390/molecules25153458)
Supplement: Supplementary file 1 [file molecules-25-03458-s001.pdf]

Supplementary Materials

A Flavone-based Solvatochromic Probe with Low Perturbation Impact on the Membrane Physical State

Simona Concilio [1,*], Miriam Di Martino [2], Anna Maria Nardiello [2], Barbara Panunzi [3], Lucia Sessa [2], Ylenia Miele [4], Federico Rossi[5], and Stefano Piotto [2,*]

1 Department of Industrial Engineering, University of Salerno, 84084 Fisciano, Italy;
2 Department of Pharmacy, University of Salerno, 84084 Fisciano, Italy;
3 Department of Agriculture, University of Napoli Federico II, 80055 Portici, Italy
4 Department of Chemistry and Biology "A. Zambelli", University of Salerno, 84084 Fisciano, Italy;
5 Department of Earth, Environmental and Physical Sciences "DEEP Sciences", University of Siena, 53100 Siena, Italy.

* Correspondence: S.C. sconcilio@unisa.it; Tel.: +39-089-964115 (S.C.), and S. P. piotto@unisa.it: Tel.: +39-089-969795.

Thickness and Deuterium Order Parameter (SCD) after the addition of the N form of the probe 3HF18

In MD simulations, double layer thickness values are typically used for the validation of lipid double layer simulations. The thickness of the lipid bilayer is calculated as the average distance between the phosphorus atoms of the two double-layer sheets. The insertion of the probe does not cause a perturbation of the membrane (Table S1). The values reported refer to the last 5 ns of the simulation.

Table S1. Thickness values (in Å) of the two membranes with and without the probe

POPC[1]	DPPC-Chol[1]	POPC[2]	DPPC-Chol[2]	POPC+3HF18(N)[3]	DPPC-Chol+3HF18(N)[4]
37.3 ± 0.3	45.9 ± 0.2	39.8 ± 0.8	44.8	37.6 ± 0.2	46.4 ± 0.2

[1] Thickness of simulated POPC and DPPC-Chol membranes; [2] Experimental thickness of POPC and DPPC-Chol membranes; [3] Thickness of POPC membrane with N 3HF18; [4] Thickness of DPPC-Chol membrane with the N 3HF18

The thickness values shown in the table indicate that the insertion of the 3HF18 N form does not cause a change in the bilayer thickness. The membrane fluidity can be typically evaluated by means of SCD (Deuterium Order Parameter) analysis. The POPC and DPPC-Chol chains were evaluated in the presence and absence of the 3HF18 probe in the N-form. Lipids around 5 Å from the molecule of interest were considered.

Figure S1 shows SCD of unsaturated chains in pure POPC membrane. The SCD decreases sharply at ~0.05 in the region near the double bond (carbon atom 9) and approaches 0 in the tail region, indicating the highest disorder within the double layer. Figure S2 shows the saturated POPC chain. It is evident that the probe in the N form does not cause a change in membrane fluidity.

The SCD for the DPPC-Chol membrane was also evaluated. Looking at Figures S3 and S4, we notice that the insertion of the probe causes a slight increase in membrane disorder.

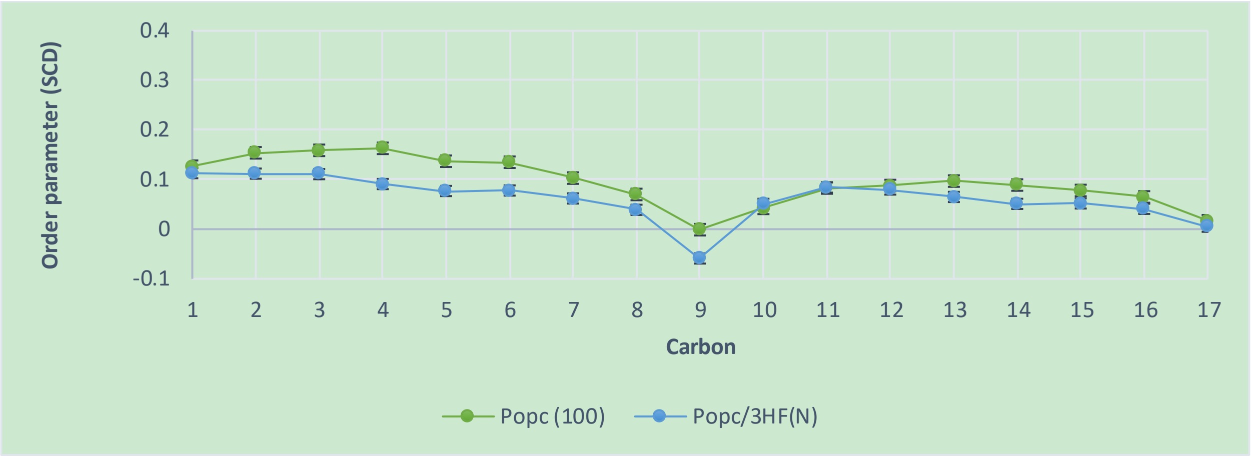

Figure S1. SCD of the unsaturated POPC chain with (blue) and without (green) the probe

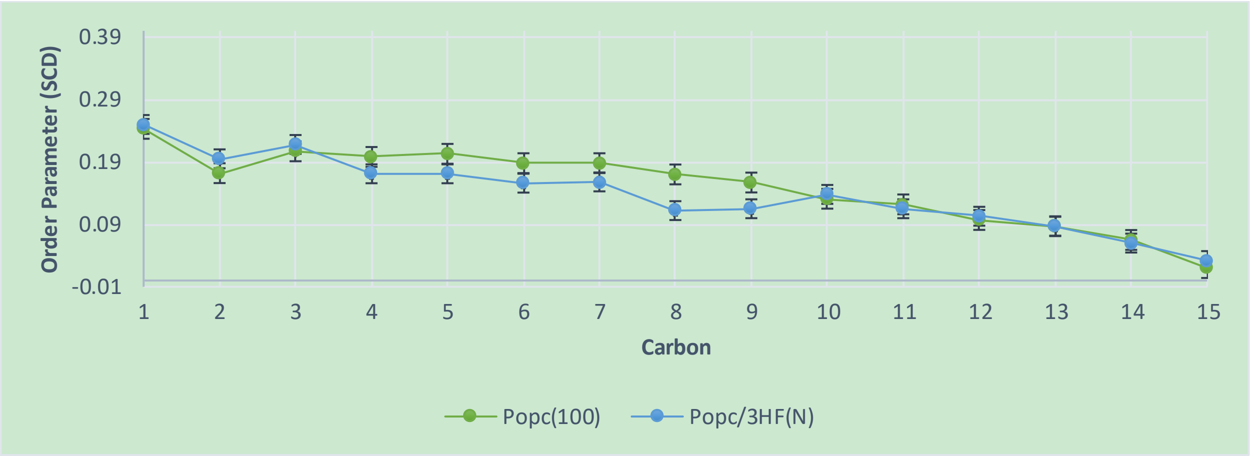

Figure S2 - SCD of POPC saturated chain with (blue) and without (green) probe

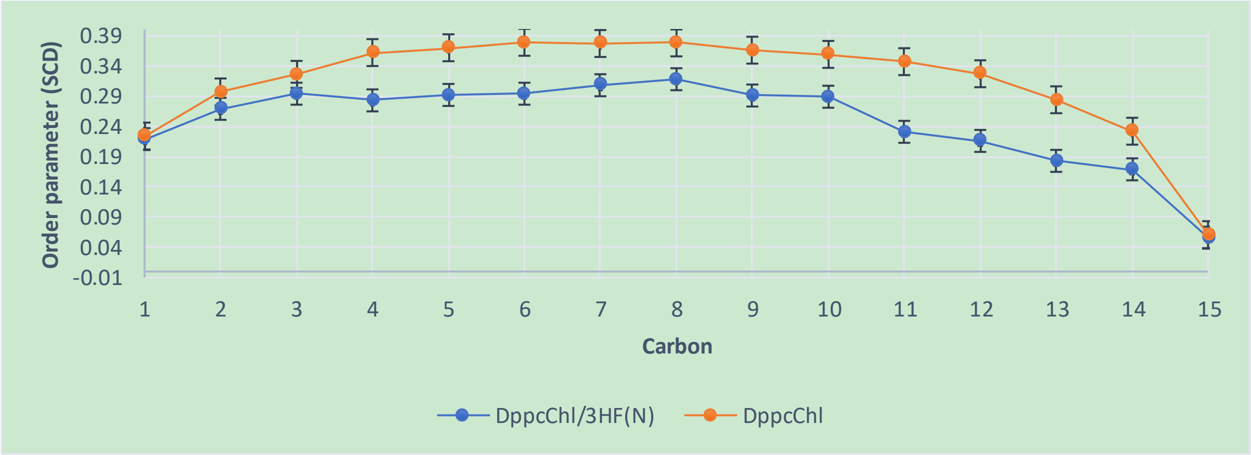

Figure S3. SCD of the DPPC Sn-1 palmitoyl chain (DPPC-Chol) with and without probe

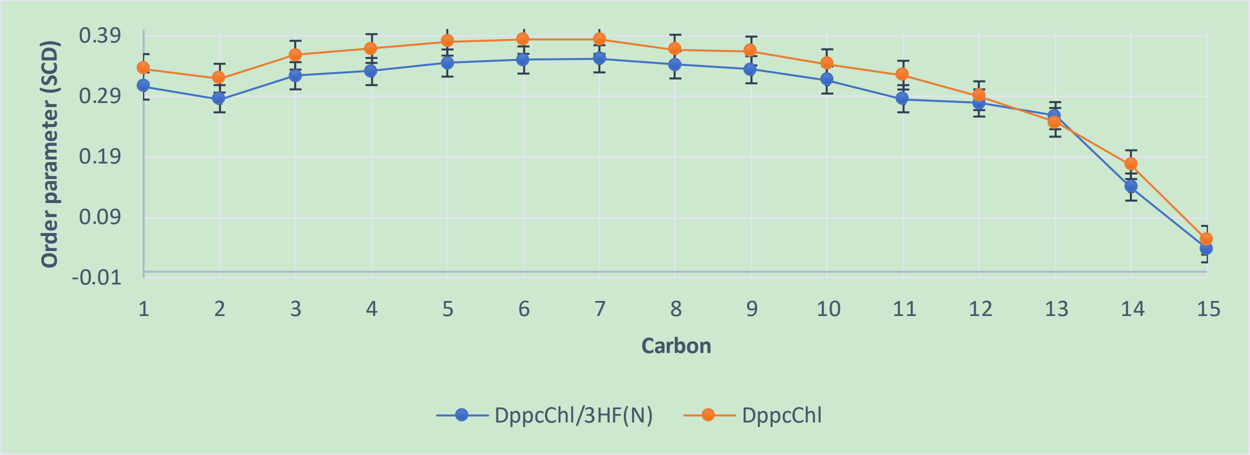

Figure S4. SCD of the DPPC Sn-2 palmitoyl chain (DPPC-Chol) with and without probe

t-Student Test

As we can see from the binding energy values reported in the work, each tautomeric form has a preference for one type of membrane: N* has a better interaction with the POPC membrane while T* interacts better with DPPC-Chol. The statistical significance of the data is validated with the t-Student test on the 4 systems. The probabilities of the null hypothesis are reported in Table S2:

Table S2. Results of t-Student test

	**p value**
N*/DppcChol – N*/Popc	0.0025
T*/DppcChol – T*/Popc	<0.0005
N*/DppcChol – T*/DppcChol	<0.0005
N*/Popc – T*/Popc	0.02

Fluorescence measurements in binary mixture MeOH/H_2O

We have also studied the solvatochromism of the new probe in a binary MeOH/H_2O mixture. As can be seen from the graph below, the fluorescence intensity decreases dramatically in the mixture due to the poor solubility of the probe in water.

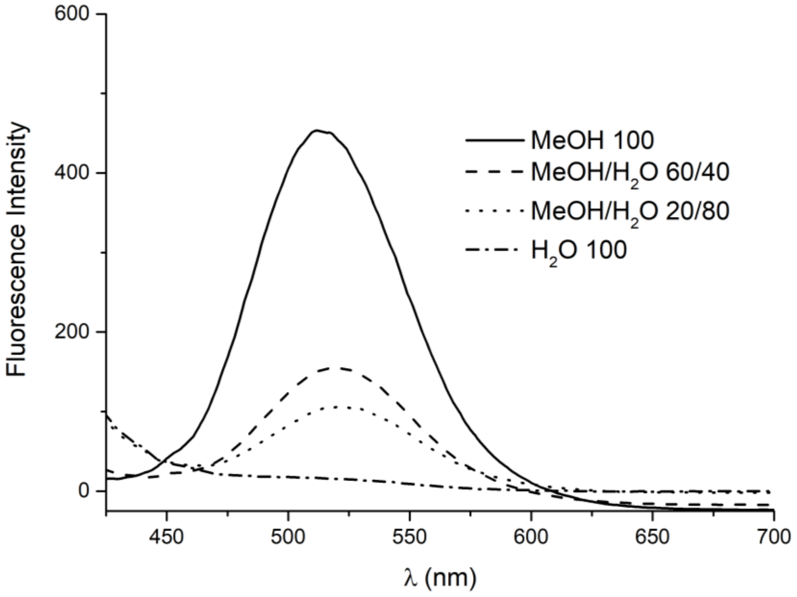

Figure S5. Fluorescence spectra of probe 3HF18 in binary mixture MeOH/H_2O in different ratios.
